# Myeloid-Derived Suppressor Cells: Implications in the Resistance of Malignant Tumors to T Cell-Based Immunotherapy

**DOI:** 10.3389/fcell.2021.707198

**Published:** 2021-07-14

**Authors:** Houhui Shi, Kai Li, Yanghong Ni, Xiao Liang, Xia Zhao

**Affiliations:** ^1^Department of Gynecology and Obstetrics, Key Laboratory of Birth Defects and Related Diseases of Women and Children, Ministry of Education, West China Second Hospital, Sichuan University, Chengdu, China; ^2^Department of Thoracic Oncology, State Key Laboratory of Biotherapy and Cancer Center, West China Hospital, Sichuan University and Collaborative Innovation Center, Chengdu, China

**Keywords:** myeloid-derived suppressor cells, T cell-based immunotherapy, combination therapy, immune checkpoint inhibitors, adoptive T cell therapy

## Abstract

T lymphocytes function as major players in antigen-mediated cytotoxicity and have become powerful tools for exploiting the immune system in tumor elimination. Several types of T cell-based immunotherapies have been prescribed to cancer patients with durable immunological response. Such strategies include immune checkpoint inhibitors, adoptive T cell therapy, cancer vaccines, oncolytic virus, and modulatory cytokines. However, the majority of cancer patients still failed to take the advantage of these kinds of treatments. Currently, extensive attempts are being made to uncover the potential mechanism of immunotherapy resistance, and myeloid-derived suppressor cells (MDSCs) have been identified as one of vital interpretable factors. Here, we discuss the immunosuppressive mechanism of MDSCs and their contributions to failures of T cell-based immunotherapy. Additionally, we summarize combination therapies to ameliorate the efficacy of T cell-based immunotherapy.

## Introduction

Immunotherapy intends to motivate immune cells to fight against tumor cells, instead of targeting tumor cells directly. The main strategies for immunotherapy include strengthening the cytotoxicity of T lymphocytes and eliminating immunosuppression induced by immune suppressive cells or factors. Notably, T cells play irreplaceable roles in the treating process for their participation in recognition of tumor antigens and final combat with tumor cells (Waldman et al., 2020). In addition, it was interpreted that T cells enrichment in tumor sites was associated with favorable responses in immunotherapy (Bruni et al., 2020). Based on this concept, multiple categories of T cell-based immunotherapy are developed and prospered.

Currently, significant strides have been made in T cell-based immunotherapy for cancer treatment since ipilimumab, an anti-cytotoxic T lymphocyte antigen-4 (CTLA-4) antibody, was approved by FDA for use in melanoma treatment. Subsequently, several categories of T cell-based immunotherapy were under active investigations or came into clinical practice (Emens et al., 2017). Such strategies are included: (i) blocking T cells suppressive signals with immune checkpoint inhibitors (ICIs) to emancipate existing T cells; (ii) reinforcing cytotoxic T lymphocytes (CTLs) through adoptive T cell therapy (ACT), such as the administration of tumor-infiltrating lymphocytes (TILs), T cell receptor-modified T (TCR-T) cells and chimeric antigen receptor T (CAR-T) cells; (iii) inducing or generating the release of tumor antigen to stimulate endogenous T lymphocytes by the application of oncolytic virus and cancer vaccines; (iv) potentiating the function of T cells by systematic administration of positive modulatory cytokines, such as interleukin (IL) and interferon (IFN). However, a number of patients quickly relapse after the initial response or fail to show any regression. Only a minority of patients benefit from these therapies (Li X. et al., 2018; Pérez-Ruiz et al., 2020). The good response cancer types include melanoma (Robert et al., 2015; Wolchok et al., 2017) and non-small cell lung cancer (NSCLC) (Antonia et al., 2017; Socinski et al., 2018). Extensive research is currently undergoing to identify determinants of resistance to obtain a superior efficacy in immunotherapy. Various factors are found to be related to the outcome of T cell-based immunotherapy: tumor mutation burden (TMB); antigen-presenting capacity; immunological characteristics of the tumor microenvironment (TME), including infiltration of effector tumor-killing cells and their counterbalance against immunosuppressive network; genetic and epigenetic alterations. In this review, we will pay emphasis on the pillar of immunosuppressive network, myeloid-derived suppressor cells (MDSCs) which often hijack T lymphocytes and abandon their cytotoxic effect on tumor cells (Colligan et al., 2020).

Myeloid-derived suppressor cell is a staple of the immune profile in the TME, which is crucial in cancer genesis, progression, and reaction to therapy (Kumar et al., 2016; Ostrand-Rosenberg and Fenselau, 2018; Tcyganov et al., 2018). In the earliest stage of tumorigenesis, cancer cells always succeed in evading immune surveillance. Once established, tumor cells release a battery of signals that induce the recruitment of immune cells from bone marrow, including MDSCs, resulting in a tumor-promoting milieu. Such is an elegant theory of immunoediting conceptualized by Robert Schreiber, whereby malignant cells, which evolve under selective pressure from immune system, gain the capability of escaping immune recognition and taking advantages of immune system in reverse (Desai et al., 2021). Moreover, therapeutic interventions, including chemotherapy, radiotherapy, and immunotherapy can simultaneously have an effect on the whole process of the TME remodeling. Apart from the direct inhibition of T cells, MDSCs can also foster the genesis and maturation of regulatory T cells (Tregs) (Veglia et al., 2018), tumor-associated macrophages (TAMs) (Ugel et al., 2015) and cancer-associated fibroblasts (CAFs) (Alkasalias et al., 2018), generating a immunosuppressive network (Tesi, 2019). Although a considerable body of original articles and reviews focus on MDSCs, few of them pay attention to their dynamic changes and the influence on T cell-based immunotherapy. Therefore, the present review attempts to figure out the signaling crosstalk to gain a comprehensive understanding of how MDSCs become immunosuppressive and how they counteract the efficacy of T cell-based immunotherapy.

## Limitation of T Cell-Based Immunotherapy

The past 10 years of clinical immunotherapy practice has engendered a deep comprehension of cancer treatment. T cells have become the central focus of arming the immune system to fight against cancer. So far, several types of T cell-based immunotherapies have been applied in clinical practice (Table 1) and nearly all the strategies augment the number of cytotoxic T cells by motivating endogenous T cells or reinfusing externally expanded T cells. A succession of biological elements needs to be fulfilled to achieve successful immunotherapy (Smyth et al., 2016). These elements include immunogenic cancer cell death, efficient antigen presentation or adjuvanticity, and active effector T cells. Notably, absence or low infiltration of cytotoxic lymphocytes was shown to be a critical inadequacy that resulted in non-response to T cell-based immunotherapy (Havel et al., 2019). Theoretically, T cell-based immunotherapies were originally designed to optimize the solution from these aspects. However, only a minority of patients experienced improved survival from these therapies. Numerous studies have been conducted to identify the barriers to a favorable response to T cell-based immunotherapy and multiple challenges have been defined.

**TABLE 1:** Summary of FDA-approved T cell-based immunotherapies.

**Therapy type**		**Formulation**	**Indicantions**
ICIs	Anti-CTLA-4	Ipilimumab	Melanoma, RCC, CRC, HCC, NSCLC, malignant pleural mesothelioma
	Anti-PD-1	Nivolumab	Melanoma, NSCLC, RCC, HL, SCCHN, Urothelial carcinoma, CRC, HCC, SCLC, ESCC, malignant pleural mesothelioma
		Cemiplimab	CSCC, NSCLC, BCC
		Pembrolizumab	Melanoma, NSCLC, SCLC, HNSCC, HL, PMLBCL, uterine cancer, bladder cancer, CRC, gastric cancer, esophageal cancer, cervical cancer, HCC, MCC, RCC, HCC, endometrial carcinoma, CSCC, TNBC
	Anti-PD-L1	Atezolizumab	Urothelial cancer, NSCLC, SCLC, TNBC, HCC, melanoma
		Avelumab	MCC, urothelial cell carcinoma, RCC
		Durvalumab	Urothelial cancer, NSCLC, SCLC
	Anti-CTLA-4 plus anti-PD-1	Nivolumab plus Ipilimumab	Melanoma, RCC, CRC, HCC, NSCLC, malignant pleural mesothelioma
ACT	CAR-T	Axicabtagene ciloleucel	NHL
		Tisagenlecleucel	ALL, NHL
		Brexucabtagene autoleucel	Mantle cell lymphoma
Oncolytic virus	Oncolytic virus	Talimogene laherparepvec	Melanoma
Cancer vaccines	DC vaccine	Sipuleucel-T	Prostate cancer
Chemokines	Interferon	Recombinant interferon alfa-2B	Hairy cell leukemia, Kaposi sarcoma, melanoma, follicular NHL
	Interleukin	Interleukin-2	RCC, melanoma

*RCC, renal cell carcinoma; CRC, colorectal cancer; HCC, hepatocellular carcinoma; NSCLC, non-small cell lung cancer; SCLS, small cell lung cancer; HL, Hodgkin lymphoma; NHL, non-Hodgkin lymphoma; SCCHN, squamous cell cancer of the head and neck; HNSCC, head and neck squamous cell carcinoma; ESCC, esophageal squamous cell carcinoma; CSCC, cutaneous squamous cell carcinoma; PMLBCL, primary mediastinal large B-cell lymphoma; MCC, Merkel cell carcinoma; TNBC, triple-negative breast cancer; ALL, acute lymphoblastic leukemia.*

Tumor immune phenotype has been identified to forecast the efficiency of immunological tumor elimination. Tumor immune classification has been proposed in recent years, and its potential value in immunotherapy was discussed in the literature. Elaborative evaluation of tumor-infiltrated lymphocytes helps to identify three predominant immune phenotypes: immune-inflamed, immune-excluded, and immune-desert (Ochoa de Olza et al., 2020). Favorable response of T cell-based immunotherapy always occurs in immune-inflamed tumors (Chen and Mellman, 2017). However, in immune-excluded and immune-desert tumors, also known as cold tumors, CD8^+^ T cells are absent or prevented from effective infiltration. Naturally, the outcome of T cell-based immunotherapy in these tumors is frustrated owing to the lack of contact-dependent cytotoxicity. Biomarkers that are correlated with tumor immune phenotype include programmed death ligand-1 (PD-L1) expression, IFN-γ signature, B cells abundance, and genetic instability, which can be used as predictors in immunotherapy (Kowanetz et al., 2018; Griss et al., 2019).

Heterogeneity of tumor cells reveals the complexity of cancer T cell-based immunotherapy. Malignant tumor is the heterogeneous product of a plethora of genetic alterations. Somatic mutation can produce tumor-specific pipetides which can be recognized by immune system as neoantigens. Also, particular molecular mutations drive the development of targeted therapies with favorable clinical response. However, tumors are as sly as foxes, always utilizing genetic alterations to escape from elimination when targeted. Therefore, although T cell-based immunotherapy has demonstrated active response, the majority of patients will eventually develop resistance to these kinds of therapies. For instance, targeting programmed death-1 (PD-1)/PD-L1 pathway triggered the upregulation of alterative immune checkpoints, such as lymphocyte-activation gene 3 (LAG3) and T cell immunoglobulin domain and mucin domain-3 (TIM-3), resulting in adaptive resistance (Koyama et al., 2016). Usually, high TMB generates antigenicity and activates immune system, making tumors inflamed. High TMB should be a positive biomarker of T cell-based immunotherapy (Samstein et al., 2019). However, recent studies did not value TMB as an effective predictor in PD-1 and CTLA-4 dual blockade therapeutic strategies (Peters et al., 2019a, b). Heterogeneity of tumor cells resulted from persistent bulky mutations may account for the incompetence of TMB in predicting benefit of immunotherapy.

Additionally, negatively regulatory networks can shut down T cell-based immune response. The TME is a dynamic environment composed of the extracellular matrix, blood vessels, signaling molecules, immune components, and malignant cells. Adding to its complicity, treatment-induced biochemical events bring about a confluence of changes. Every immune process could exert influence over the treatment outcome. Monotherapies merely manipulating T cells seem powerless to work out the major impediment that attenuates antitumor immunity. With deeper understanding of the TME and summary on clinical practice, it is consensus that the other therapeutic node, removing immune suppression, cannot be neglected (Smyth et al., 2016; Tesi, 2019).

## MDSC: A Real Setback to the Success of T Cell-Based Immunotherapy

Among the negatively regulatory networks, cellular components include Tregs, TAMs, CAFs, and MDSCs. They secrete a plethora of cytokines and chemokines, providing hotbed and protector for cancer cells. Notably, MDSCs are acknowledged to directly compromise T cells function, hence their name. Accumulating evidence has suggested the vital role of MDSCs in facilitating an immunosuppressive TME in various cancer types (Parker et al., 2015; Kumar et al., 2016). Numerous immunosuppressive mechanisms have been identified, including soluble mediators, metabolic interactions, and cell-to-cell contact.

### MDSCs: A Heterogeneous Clump of Immature Myeloid Cells

Myeloid-derived suppressor cells are a diversified clump of immature myeloid cells with strong immunosuppressive functions. Generally, most MDSCs could fall into two categories, namely: monocytic-MDSCs (M-MDSCs) and polymorphonuclear-MDSCs (PMN-MDSCs, also known as granulocytic-MDSCs, G-MDSCs). In humans, scientists also defined a more immature MDSC as early-stage MDSC (e-MDSC), lacking surface markers for both M-MDSC (CD14^+^) and PMN-MDSC (CD15^+^) (Bronte et al., 2016). Under homeostatic conditions, hematopoietic stem cells can polarize towards mature granulocytes, monocytes or dendritic cells (DCs) through common myeloid progenitors (CMPs) and granulocyte-monocyte progenitors. Nevertheless, under pathologic conditions such as chronic infection, cancer or immune-related disease, granulopoiesis is compromised, diverting the mainstay differentiation towards MDSCs (Mackey et al., 2019). Recently, conversion of neutrophils and monocytes to MDSCs has been reported. In particular, CD14^+^ myeloid cells isolated from melanoma cells have been demonstrated to develop a suppressive activity on T cells when disposed with extracellular vesicles (EVs), thus referred to as EV-MDSCs. Similarly, when exposed to exosomes obtained from chronic lymphocytic leukemia (CLL) cells, monocytes derived from healthy donors displayed the functional characteristics of MDSCs (Bruns et al., 2017). However, Bronte et al. (2016) suggested that human MDSCs generated *in vitro* should be defined as “MDSC-like” cells. A separate team also identified a monocytic lineage as the precursor of PMN-MDSCs and termed these cells monocyte-like precursors of granulocytes (Mastio et al., 2019). Intriguingly, selective inhibition of monocytic cells had little impact on the genesis of granulocytes in normal mice although it diminished the aggregation of PMN-MDSCs by 50% in tumor-bearing mice. All these findings showed that MDSCs are generated in a complicated hematopoietic network, in which multiple hematopoietic cells have the potential to differentiate into to MDSCs and this process is stringently regulated by factors derived from the TME.

The MDSCs expansion and activation are controlled by multiple TME-derived molecules *via* several transcriptional pathways. Growth factors such as the stem cell factor (SCF), granulocyte colony-stimulating factor, macrophage colony-stimulating factor, and granulocyte-macrophage colony-stimulating factor (GM-CSF), stimulate myelopoiesis and expansion of MDSCs through JAKs-STAT signaling pathways (Kumar et al., 2016). Among several STAT signaling pathways, STAT3 was the most widely studied and was shown to promote MDSCs proliferation by regulating the expression of proliferation-related genes and apoptosis-related genes, such as bcl-xl, cyclin d, and c-myc (Wang et al., 2020). STAT3 was also reported to induce the production of immunosuppressive factors such as arginase 1 (ARG1) and reactive oxygen spices (ROS) (Wang et al., 2020). In addition, inflammation is another factor affecting the modulation of MDSCs. Inflammation is considered as a companion of cancer and is well involved in nearly all stages of cancer development and the TME sculpture (Greten and Grivennikov, 2019). In the inflammatory milieu, MDSCs are generated to prevent the immune system from overreacting. While get into a cancer-related inflammatory milieu, MDSCs are quickly tamed by cancer cells and transform into a tumor-protective phenotype (Bronte et al., 2016). Inflammatory cytokines, including S100A8/9, adenosine, IFN-γ and IL-6 are responsible for the remodeling of MDSCs by activating toll-like receptor (TLR) signaling pathway (Wang et al., 2020). Furthermore, transforming growth factor-β (TGF-β) and IL-1β were reported to restrain the transcription of human leucocyte antigen-DR (HLA-DR) in monocytes by dampening major histocompatibility complex class II (MHC II) transactivator, converting them to CD14^+^HLA-DR^lo/neg^ M-MDSCs (Mengos et al., 2019). Additionally, the CCAAT/enhancer binding protein-α (C/EBPα) is an extensively engaged transcription factor that is also imperative for the function of MDSCs (Thevenot et al., 2014). C/EBPα drives the expansion of MDSCs through a cell-surface molecule called the retinoic acid-related orphan receptor C1, during cancer-related inflammation (Strauss et al., 2015). Moreover, the C/EBP homologous protein is an apoptosis-related transcription factor which is stimulated by endoplasmic reticulum (ER) stress and is instrumental in the activation of the IL-6/STAT3 pathway in MDSCs. On the contrary, interferon regulatory factor-8 (IRF8) negatively regulates of the generation of MDSCs, on the account that transgenic overexpression of IRF8 diminished the aggregation of MDSCs and mice with low expression of IRF8 were enriched with MDSC-like cells (Valanparambil et al., 2017).

Additionally, ER stress has emerged as a key mechanism regulating the pathologic activities of MDSCs. MDSCs from tumor-bearing hosts exhibit a greater ER stress response than monocytes or neutrophils from tumor-free hosts (Condamine et al., 2014). Neutrophils derived from healthy donors develop a suppressive capacity when exposed to inducers of ER stress (Condamine et al., 2014). These experimentally induced cells expressed high levels of ARG1, NOS2, and NAPDH oxidase-2 (NOX-2), which are closely associated with immunosuppression. Moreover, ER stress regulates the lifespan of MDSCs, favoring their apoptosis in peripheral tissues and promoting their expansion in bone marrow (Condamine et al., 2014). An additional study showed that thapsigargin-induced ER stress upregulated the level of the lectin-type oxidized LDL receptor 1 (LOX-1) in human neutrophils and promoted their conversion to the immunosuppressive phenotype, which can be interrupted by blocking the inositol-requiring enzyme 1-spliced X-box binding protein-1 pathway (Condamine et al., 2016). Nonetheless, it is still unclear whether LOX-1 is important in the downstream signal transduction to prompt neutrophils to acquire a suppressive potential.

The accumulation of MDSCs in the tumors sites is regulated by chemokines generated from the TME. C-C motif chemokine ligand 2 (CCL2, also called MCP-1) and CCL5 (also called RANTES) are the main chemotactic factors implicated in the migration of M-MDSCs into tumors (Iwamoto et al., 2020). Increased expression of CCL2 is an indicator of the progression of human colorectal cancer while inhibiting CCL2 was reported to reduce the recruitment of MDSCs in colorectal cancer model (Chun et al., 2015). Similarly, PMN-MDSCs are mobilized following the chemotaxis of CXC motif chemokines, including CXC motif chemokine ligand 1 (CXCL1), CXCL2, CXCL5, CXCL8, and CXCL12 (Kumar et al., 2016; Najjar et al., 2017; Li Y.M. et al., 2019). S100A8 and S100A9 proteins are also among the critical driving force in MDSCs recruitment (Song and Struhl, 2021). Notably, PMN-MDSCs are also the sources of S100 proteins, creating a positive feedback loop of MDSCs aggregation (Kowanetz et al., 2010). Evidently, the recruitment of MDSCs involves complicated mechanisms and it occurs according to the type and stage of tumors.

### MDSC: A Critical Role the Immunosuppressive Camp

If the immune cells within the TME were divided into two opposing fronts, T cells would be considered as the leaders of the friendly side while MDSCs occupy critical position in the pernicious foe (Tesi, 2019). MDSCs deliver suppression on cytotoxic lymphocytes and innate immune cells. Additionally, they exert their effects on other inhibitory cell populations, forming a powerful immunosuppressive landscape and inhibiting the tumoricidal immune system.

#### Construction of an Immunosuppressive Camp

**FIGURE 1:**
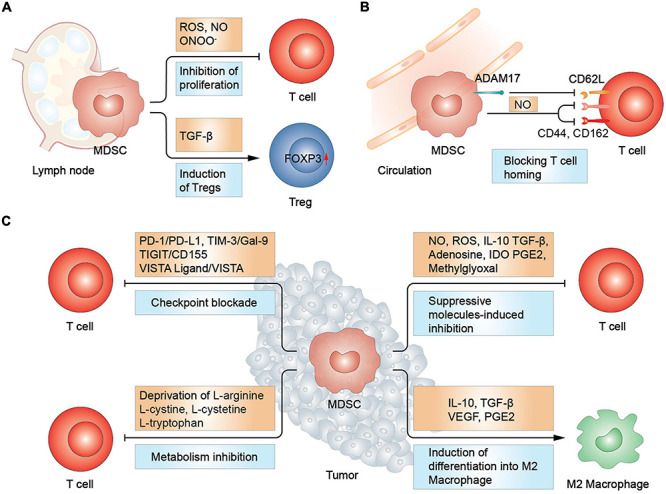
MDSCs-mediated suppression in T cells. **(A)** In lymph node, MDSCs promote Treg differentiation and increase FOXP3 expression *via* TGF-β. T cell proliferation was inhibited due to the increased secretion of reactive oxygen and nitrogen spices. **(B)** MDSCs inhibit T cell homing by disrupting L-selectin (CD62L) structure and hindering the function of CD44 and CD162, essential in T cell homing. **(C)** In tumor sites, MDSCs inhibit T cells *via* immune checkpoint blockade, metabolism deprivation, and suppressive molecules. Moreover, MDSCs induce macrophage differentiation into type 2 macrophage (M2 macrophage). Treg, regulatory T cell; IDO, indoleamine 2,3-dioxygenase.

During the battle against cancer cells, Tregs and type 2 macrophages betrayed the T cells camp and became accomplices of the MDSCs. When co-incubated with M-MDSCs separated from pancreatic ductal adenocarcinoma, autologous CD4^+^ T cells could potentiate their differentiation to Foxp3^+^ Tregs (Siret et al., 2020). Th17 cells were also revealed as a source of Foxp3^+^ Tregs under the induction of CD14^+^HLA-DR^–/low^ M-MDSCs *via* TGF-β and retinoic acid (Hoechst et al., 2011). Furthermore, MDSCs-derived molecules, such as CCL3, CCL4, and CCL5, facilitate the recruitment of CCR5^+^ Tregs in a mouse model of melanoma (Schlecker et al., 2012). Moreover, a recent preclinical study demonstrated the MDSCs-mediated induction of Tregs occurred in a cell contact manner (Siret et al., 2020). In parallel with mobilization of Tregs, MDSCs can also convert macrophages to type 2 phenotype through releasing increased level of IL-10, thereby promoting tumor progression (Zefferino et al., 2021). More importantly, it was shown that M-MDSCs could directly differentiate into TAMs in a hypoxia inducible factor-1α (HIF-1α) dependent manner (Zefferino et al., 2021). Th17 cells are another group of immunosuppressive cells characterized by the production of IL-17, which cripple T lymphocyte cytotoxicity. MDSCs induce inducible nitric oxide synthase (iNOS) production in T cells, favoring Th17 cells proliferation (Jayakumar and Bothwell, 2019; Dar et al., 2020). Conversely, Tregs were also observed to contribute to the development of MDSCs. Lee et al. (2016) showed that Tregs facilitated the proliferation of MDSCs through the secretion of TGF-β in a colitis model. The study also reported that reduced secretion of TGF-β impaired the expression of ARG1, iNOS, and PD-L1 in M-MDSCs. In a mouse ret melanoma model, Tregs from the tumor site not only inhibited T cells directly but also upregulated PD-L1 expression in MDSCs. Correspondingly, deletion of Tregs using CD25-specific antibodies downregulated the expression of PD-L1, suggesting an interlaced relationship between MDSCs and Tregs (Fujimura et al., 2012). Overall, these findings verified the presence of a powerful negative regulatory network comprising of MDSCs and other inhibitory immune cells, conspiring to abrogate T cell-based antitumor immune response and promote tumor progression (Figure 1).

#### Suppression on T Cells

Myeloid-derived suppressor cells are major sources of reactive oxygen and nitrogen spices in the TME, which are harmful to most cell types including T cells. The production of ROS by NOX-2 during respiratory burst, such as hydrogen peroxide (H_2_O_2_), superoxide anions and hydroxyl radicals, was found to be closely related to the eradication of TILs. ROS inhibited T-cell immune responses by restricting interactions between TCR and MHC, whereas ROS inhibitors could reverse the suppressive effect of MDSCs on T cells (Liu et al., 2015). Moreover, elevated ROS levels suppressed their differentiation to mature myeloid cells, enhancing the recruitment of MDSCs and forming a signaling cascade (Adeshakin et al., 2021). Intriguingly, MDSCs were demonstrated to salvage the harmful effect of ROS by expressing the nuclear factor erythroid 2-related factor 2 (Nrf2), an transcription factor mediating cellular antioxidant response (Beury et al., 2016). Another cytotoxic oxynitrite is nitric oxide (NO), which is produced through the catalysis of iNOS. NO was declared to suppress proliferation of T lymphocytes and even prompt their apoptosis. Moreover, its synthesis products with the superoxide anion, peroxynitrite, should be responsible for non-responsiveness of CTLs.

Furthermore, MDSCs deprive T cells of several nutrients such as L-arginine and cysteine which are essential for their metabolism and function (Geiger et al., 2016). Cationic amino-acid transporter-2B works as a mediator in the transportation of L-arginine from the extracellular space into the intracellular space of MDSCs (Cimen Bozkus et al., 2015). A previous study reported that when L-arginine was exhausted by MDSCs, nitration of TCRs and T-cell apoptosis were enhanced through the increased production of IL-10 and prostaglandin E2 (PGE2), leading to impairment T cells activation (Miret et al., 2019). When gathered in MDSCs, L-arginine serves as a substrate in a cascade of synthetic reactions, contributing the immunosuppressive function of MDSCs (Grzywa et al., 2020). For instance, arginases (ARG1 and ARG2) deal with the catabolism of L-arginine to urea and L-ornithine. L-ornithine can further convert to L-proline which is an important immunosuppressive polyamine (Grzywa et al., 2020). Moreover, activated T lymphocytes, together with several Th2 cell-secreted cytokines, including IL-4, IL-5, IL-10, and IL-13 can increase the expression of ARG1 (Grzywa et al., 2020). Upregulation of ARG1 in MDSCs not only trigger T cells inhibition but also promotes extracellular matrix remodeling, favoring tumor growth (Grzywa et al., 2020). However, it was recently demonstrated that the expression of ARG1 is not compulsory in MDSCs-mediated immunosuppression (Bian et al., 2018). The study however emphasized on the importance of cell-to-cell contact in T cells inhibition. Cysteine is another essential amino acid in T cells that was also reported to be deleted by MDSCs, leading to the inhibition of T-cell function (Srivastava et al., 2010). It was shown that depletion of L-arginine and cysteine reduced the synthesis of glutathione (GSH), which could prevent the production of ROS (Srivastava et al., 2010). In addition to amino acid metabolism, alteration of glycolysis and lipid metabolism contributes to MDSCs inhibitory capability, resulting in T cells suppression and tumor progression (Wang et al., 2020). The increased production of lactate augmented the number of MDSCs and fatty acid transport protein 2 was reported to be overexpressed in PMN-MDSCs, which could exert suppressive function by means of arachidonic acid uptake and synthesis of PGE2.

Immunosuppressive mediators are also utilized by MDSCs to induce T cells inhibition. In cancer-bearing hosts, adenosine is excessively produced in the TME. CD39 and CD73 are two critical ectoenzymes that catalyze the generation of adenosine and are mainly expressed on immunosuppressive cells. MDSCs separated from the peripheral blood or tumors of NSCLC patients were reported to express increased levels of CD39 and CD73, under the stimulation of TGF-β in a HIF-1α-dependent way, hence resulting to excessive production of adenosine (Li et al., 2017). Additionally, adenosine was proven to inhibit naïve T cells initiation by blocking the phosphorylation of extracellular regulated protein kinases (ERK), protein kinase B (PKB/Akt) and the zeta-chain-associated protein 70 (Zap70) (Mastelic-Gavillet et al., 2019). The function of activated T cells was also compromised by adenosine, which attenuated the expression of several effector molecules such as CD95L, perforin, IFN-γ, and tumor necrosis factor-α (TNF-α) (Mastelic-Gavillet et al., 2019). Apart from high levels of adenosine, other immunosuppressive cytokines (such as IL-10 and TGF-β) from MDSCs also elicit T cells inhibition. For instance, IL-10 inhibits antigen presentation by impairing the expression of MHC, co-stimulatory signals and cytokines secretion in antigen presenting cells (APCs), hence indirectly suppressing T cells response (Payne et al., 2013). Furthermore, TAMs, TILs, as well as cancer cells are also the source of IL-10 and TGF-β, amplifying their tumor promoting and tumor suppressing activity (Stewart and Smyth, 2011). Therefore, successful treatment requires more specific identification of tumor immune signatures when targeting IL-10 and TGF-β. In addition, PGE2 and indoleamine 2,3-dioxygenase (IDO) were also involved in MDSCs-induced immunosuppression. In a genesis assay of MDSCs, PGE2 was shown to induce the expression of multiple immunosuppressive makers on MDSCs, including IDO, IL-10, and ARG1 (Obermajer and Kalinski, 2012).

Moreover, immune checkpoint molecules were identified to be strong mediators of MDSCs-induced immunosuppression. PD-1/PD-L1 is the mostly studied signal. The binding of PD-L1 on MDSCs and PD-1 on T cells induces T cells anergy and apoptosis (Groth et al., 2019). Moreover, other immune checkpoints are also involved in MDSCs-induced suppression on T cells, including CTLA-4, LAG-3, and TIM-3. These several immune checkpoints complement each other. For instance, the TIM-3/Gal-9 pathway is a critical mechanism for primary or secondary resistance to anti-PD-1 treatment in metastatic NSCLC patients (Limagne et al., 2019). In addition, in patients with high MDSCs levels, ICIs have poorer clinical response. So, whether ICIs could interrupt immune checkpoint-mediated connections between MDSCs and T cells need further investigation.

In addition to the aforementioned mechanisms, MDSCs also impair lymphocytes homing by destroying the structure of CD62L (L-selectin), a lymph node homing receptor, through a disintegrin and metalloprotease 17 (ADAM17) (Ku et al., 2016). The loss of L-selectin was also proved to be correlated with inactivation of CD8^+^ T cells in lymph nodes (Ku et al., 2016). Further, M-MDSCs could destroy the structure of trafficking-related molecules on T cells in a NO dependent manner, including CD44 and CD162 (Schouppe et al., 2013). CD44 mediates the connection of T cells with extracellular matrix component hyaluronic acid and CD162 is a ligand of selectin P. The destroy of CD44 and CD162 impaired the extravasation and the infiltration of T cells. MDSCs set the myriad of hurdles in T cells trafficking way.

Most studies of immunotherapy concentrated on conventional CD4^+^ and CD8^+^ lymphocytes, but unconventional T cells may offer advantages to T cells immunotherapy in cancer treatment, which are also susceptible to MDSCs. The unconventional T cells subsets include natural killer T (NKT) cells, mucosal-associated invariant T (MAIT) cells and γδ T cells (Pellicci et al., 2020). These cells account for approximate 10% of T cells in circulation and a dominant percentage in tissues such as gut mucosa. Conventional T cells functions in a MHC I or MHC II dependent manner, while unconventional T cells interact with MHC class Ib or MHC I like molecules (Godfrey et al., 2018). Activation of type I NKT cells was shown to stimulate the motivation of T cells and NK cells, while the behavior of type II NKT cells was drastically different (Vivier et al., 2012). At the presence of α-GalCer, type I NKT cells could convert MDSCs into stimulatory APCs by producing increased levels of IL-12, IFN-γ, and TNF (Ko et al., 2009). In turn, Zhang et al. (2017) reported that MDSCs could selectively inhibit the production of IFN-γ of NKT cells through membrane-bound TGF-β. However, other two studies revealed that NKT cells were resistant to immunosuppressive effects of MDSCs (Gebremeskel et al., 2015; Horinaka et al., 2016). The quite different observations may be caused by discrepant identifying markers. More sophisticated design is needed to achieve an accurate assessment. γδ T cells also have dual effects on tumors, which is dependent on the characteristics of TME (Li L. et al., 2019). Under normoxic environment, tumor-derived exosomes stimulate the cytotoxic activity of γδ T cells (Li L. et al., 2019). However, oxygen pressure could alter the content of tumor-derived exosomes, which subsequently induced the suppression of MDSCs on γδ T cells (Li L. et al., 2019). Additionally, a subset of Vγ4 γδ T cells are the producer of IL-17, an immune inhibitory cytokine. This subset of Vγ4 γδ T cells enhance the suppressive activity of MDSCs, forming a positive feedback (Ma et al., 2014). The diversity of unconditional T cells and limited identification strategies pose challenge to clearer understanding of their biological characteristics and targeted strategies.

#### Impact on Other Tumor Inhibitory Immune Cells

Apart from direct T-cell suppression, MDSCs also exert strong regulatory effects on other tumor inhibitory immune cells, such as NK cells and DCs, hence generating a formidable immunosuppressive force (Ostrand-Rosenberg et al., 2012; Bruno et al., 2019). M-MDSCs obtained from melanoma patients inhibited the cytotoxicity of NK cells through the production of TGF-β (Mao et al., 2014). The same study showed that monocytes treated with PGE2 were activated *via* the p38MAPK/ERK pathway and consequently secreted elevated levels of TGF-β, resulting in the potent suppression of NK cell activity *in vitro*. In another study, both CD14^+^ MDSCs and CD15^+^ MDSCs from the tumors of head and neck squamous cell carcinoma (HNSCC) patients suppressed NK cells activation (Greene et al., 2020). Moreover, the immunosuppressive ability of CD14^+^ MDSCs was reversed using TGF-β monoclonal antibodies (mAb) while CD15^+^ MDSCs were only sensitive to the NOS inhibitor, L-NMMA. In addition to soluble factors, Fc receptors expressed on NK cells were also reported to be critical in MDSCs-induced suppression (Stiff et al., 2018).

Increasing evidence suggests that MDSCs are associated with disrupting the accumulation and function of DCs, although the underlying mechanism is yet to be elucidated. Poschke et al. (2012) demonstrated that purified M-MDSCs extracted from melanoma patients negatively regulated the maturation, trafficking, cytokine production and antigen presentation of DCs. Additionally, in hepatocellular carcinoma model, MDSCs suppressed the capability of DCs to stimulate T lymphocytes and inhibited IL-12 production in DCs through increased IL-10 secretion (Hu et al., 2011). A recent study also demonstrated that MDSCs hindered DCs-mediated antigen presentation, which was dependent on NO-induced STAT1 nitration, and this effect could be reversed by iNOS inhibitors (Markowitz et al., 2017). Moreover, a previous study reported that MDSCs suppressed the generation and antigen presentation of DCs. It also showed that both Notch and STAT3 signaling were required in MDSCs-induced suppression (Wang et al., 2016).

Additionally, MDSCs are involved in B cell-mediated immune responses. A study reported that MDSCs impeded B cells proliferation *in vitro* through the increased secretion of ARG1 (Wang et al., 2018). After deletion of MDSCs using anti-Gr-1 antibodies, there was an increase in the production of both IL-7 (a B cell stimulator) and B cell-derived IgG, indicating that B cells suffer from MDSCs-induced inhibition. Similar to T cells, Ku et al. (2016) announced that MDSCs also reduced L-selectin levels on B cells through the cell-to-cell contact mechanism, hence disrupting the homing of B cells. Furthermore, it was shown that MDSCs upregulated the expression of PD-L1 and IL-10 on IgA^+^ B cells *via* the TNF receptor 2 (Xu et al., 2017). Moreover, MDSCs act as inducers of regulatory B cells, a cluster of immunosuppressive B cells, by delivering IL-10 and TGF-β (Jayakumar and Bothwell, 2019).

In addition to their immunosuppressive capabilities, MDSCs also positively contribute to the immune-independent progression of tumors. They promote the expansion of tumor cells, epithelial-mesenchymal transition (EMT), and enhance stemness by secreting numerous soluble factors, such as IL-10 and TGF-β (Yang et al., 2004; Bruno et al., 2019). Moreover, MDSCs were shown to facilitate metastasis by secreting metalloproteases, such as matrix metallopeptidase 9 (MMP-9), and contribute to pre-metastasis niche formation (Long et al., 2020). When get into a pre-metastasis niche, MDSCs release a large amount of NO, ARG-1, and immunosuppressive factors, altering T cells and NK cells functions and facilitating the recruitment of TAMs and Tregs (Tang et al., 2021). Additionally, MDSCs promote angiogenesis by secreting MMP9 and VEGF (Tang et al., 2021).

### MDSC: An Adverse Predictive Marker in Cancer Patients

Myeloid-derived suppressor cells have been determined in multiple kinds of tumors, such as melanoma, NSCLC, and prostate cancer. Studies on patients payed more attention to MDSCs in peripheral blood, contrary to preclinical studies which largely focused on tumor-infiltrating MDSCs. MDSCs accumulation in circulation always coincides with an advanced tumor burden, stage, grade, and poor prognosis in various types of cancer (Diaz-Montero et al., 2014). For instance, higher percentages (>11%) of circulating CD14^+^ M-MDSCs independently predicted the risk of death in stage III/IV melanoma patients (Weide et al., 2014). A similar conclusion was also drawn in patients with NSCLC, pancreatic cancer, bladder cancer, gastric cancer, and hepatocellular carcinoma (Chaib et al., 2020).

The MDSCs abundance in circulation was also verified to be correlated with poorer responses to various types of immunotherapy and they shortened the overall survivals (OS) of patients (Gebhardt et al., 2015; Martens et al., 2016). Meyer et al. (2014) reported that low frequencies of M-MDSCs were related to better clinical efficacy in melanoma patients treated with ipilimumab. This finding was consistent with that of another study which reported that high levels of MDSCs dealt with the absence of antigen-specific T lymphocytes, hence engendering limited efficacy of ipilimumab (Weide et al., 2014). Three more studies also reported that lower levels of circulating MDSCs before treatment could be utilized as a predictive marker of favorable reaction to ipilimumab in melanoma patients (Gebhardt et al., 2015; Martens et al., 2016). A similar pattern was also observed in melanoma patients undergoing anti-PD-1 treatment. Fewer pretreatment MDSCs in peripheral blood were associated with better clinical responses and longer survival of melanoma patients treated with nivolumab (Weber et al., 2016). Furthermore, there was an increase in the levels of LOX-1^+^ PMN-MDSCs (a specific subset of MDSCs) following anti-PD-1 treatment in NSCLC patients with hypo-responsiveness (Kim et al., 2018). LOX-1 was identified as a promising marker for distinguishing between immunosuppressive PMN-MDSCs and neutrophils (Condamine et al., 2016). The accumulation of LOX-1^+^ PMN-MDSCs in tumor site was also correlated with shorter disease free survival in glioblastoma patients (Chai et al., 2019). These findings therefore indicate that MDSCs always appear to cripple the beneficial effects of immunotherapy by interfering with the function of T cells.

### Effect on T Cell-Based Immunotherapy

T cells play irreplaceable roles in immunotherapy. Efficient antigen presentation, sufficient activation and expansion, smooth infiltration, and effective cytotoxicity of T cells are indispensable in successful immunotherapy. Given the mechanism of MDSCs-mediated immunosuppression has been widely studied, their effect on clinical response of T cell-based immunotherapy can never be neglected.

#### Effect on Immune Checkpoint Inhibition

Immune checkpoint inhibitors have gained popularity owing to their efficacy in several malignant tumors (Darvin et al., 2018). The PD-1/PD-L1 axis and the CTLA-4/B7 axis are key targets for ICIs and have been widely applied in clinical practice (Tang et al., 2018; Melaiu et al., 2020). Ipilimumab, the first approved anti-CTLA-4 monoclonal antibody, slightly but significantly increased the OS of stage III/IV melanoma patients compared to standard treatments (Hodi et al., 2010). Thereafter, nivolumab and pembrolizumab were approved by the FDA in 2014. The drugs were shown to have durable responses in 40% of patients and conferred an improved OS, compared to chemotherapy and ipilimumab (Robert et al., 2014, 2015). Following this, anti-PD-L1 antibodies, including atezolizumab, avelumab, and durvalumab along with another anti-PD-1 antibody were gradually approved by the FDA, with extensive indications on several types of cancer (Gulley et al., 2017; Hassan et al., 2019). Unfortunately, trials on ipilimumab in several cancer types, such as NSCLC, SCLC, renal cell carcinoma, and prostate cancer did not provide results as satisfactory as those seen in patients with melanoma (Lynch et al., 2012; Reck et al., 2013; Kwon et al., 2014). Additional trials on PD-1/PD-L1 inhibitors, such as atezolizumab, showed little advantage over standard treatment in certain kinds of solid tumors (Powles et al., 2018).

Based upon the success of the PD-1/PD-L1 and CTLA-4/B7 blockade, additional immune checkpoints are extensively being explored inpreclinical and clinical investigations (Andrews et al., 2019). LAG3, TIM-3, T cell immunoreceptor with immunoglobulin and the immunoreceptor tyrosine-based inhibitory motif domain (TIGIT) are alternative inhibitory receptors that dampen T cell functions by binding to their ligands (Anderson et al., 2016). LAG3, expressed on T cells, NK cells, plasmacytoid DCs and B cells, is among the most heavily studied targets other than PD-(L)1 and CTLA-4 (Maruhashi et al., 2020). The binding of LAG3 to its ligand, MHC II, causes the suppression of effector T cells, inhibiting their proliferation and effector activities (Huard et al., 1994). Currently, various agents targeting LAG-3 are in the clinical trial either as monotherapies or supplement of anti-PD-(L)1 treatment, but the efficacy is limited (Hong et al., 2018; Maruhashi et al., 2020). TIM-3 is also expressed on several immune cell types, such as T cells, Tregs, DCs, and NK cells, modulating their functions by binding to numerous ligands (Wolf et al., 2020). For instance, binding to galectin 9 induces T cells death and binding to the carcinoembryonic antigen cell adhesion molecule 1 (CEACAM1) appears to induce immune tolerance. In addition, overexpression of TIM-3 predicts poor outcome in multiple cancer types and anti-TIM-3 was shown to reduce suppression on IFN-γ-producing CD8^+^ T cells (Fourcade et al., 2010; Lu et al., 2017b; Shayan et al., 2017). Early phase clinical trials on the anti-TIM-3 antibody, did not show active data (Curigliano et al., 2019; Harding et al., 2019). TIGIT is an inhibitory receptor of the immunoglobulin superfamily, participating in the restriction of both adaptive and innate immune responses (Andrews et al., 2019; Chauvin and Zarour, 2020). TIGIT is expressed principally on T-cell subsets and NK cells (Joller et al., 2011). However, previous research showed that a single TIGIT blockade had no real benefit in CT26 tumor models while combination therapy with the PD-1/PD-L1 blockade resulted in complete tumor rejection through augmenting of CD8^+^ T cells proliferation and function (Johnston et al., 2014). Similar results was observed in patients with advanced solid tumors treated with anti-TIGIT antibody, MK-7684, showing 8 partial responses out of 43 individuals in anti-PD1 combination group (33rd Annual Meeting & Pre-Conference Programs of the Society for Immunotherapy of Cancer (SITC, 2018), 2018). In addition to these inhibitory targets, several other “second generation” immune checkpoints are currently under intense investigation, including the V-domain immunoglobulin suppressor of T cell activation (VISTA), B7-H3 (CD276) as well as the B- and T-lymphocyte attenuator (BTLA, CD272) (Andrews et al., 2019). However, given that nearly all these targets came up recently, the preliminary efficacy and safety of related targeting strategies are not known.

While several studies have reported that ICIs have positive clinical outcomes, most patients with advanced cancers, accounting for a significant proportion of cancer-related deaths, have not experienced substantial cancer regressions or improved survival after the treatments (Topalian et al., 2012; Callahan et al., 2018). Only 20% of unselected NSCLC and 40% of melanoma patients respond to PD-1/PD-L1 blockade (Brahmer et al., 2012; Shukuya and Carbone, 2016). Numerous studies targeting LAG-3, TIM-3, TIGIT, VISTA, B7-H3, and BTLA, mainly in early phase, are carrying out, but to little avail. Combination therapy with a conventional strategy or another immunotherapy could be an effective method (Gandhi et al., 2018). However, it is difficult to obtain substantial efficacy due to the non-response or acquired resistance to ICIs.

Several reports have shown that MDSCs promote ICIs resistance. A prospective cohort study of NSCLC patients showed a decreased number of M-MDSCs in the nivolumab-response group whereas the data of non-responders stayed steady. *In vitro*, researchers found resuscitated secretion IFN-γ of CD8^+^ T cells under treatment of anti-PD-1, which was impaired by M-MDSCs *via* the bond of galectin-9 and its receptor TIM-3, indicating that MDSCs can hinder the function of T cells (Wolchok et al., 2017; Limagne et al., 2019). When treated with ipilimumab, responders showed a remarkably lower percentage of M-MDSCs than non-responders (Meyer et al., 2014).

The baseline of MDSCs are associated with ICI response. Weber et al. (2016) found that patients with lower initial M-MDSCs levels in circulation had a better response to nivolumab. This concept was also detected in melanoma patients under nivolumab or ipilimumab treatment (Gibney et al., 2015; Bjoern et al., 2016). Melanoma patients with low baseline MDSC had 34.5% survival benefit compared to patients from higher initial MDSC level group. A computational algorithm was used to analyze the role of peripheral blood mononuclear cells (PBMCs). According to the program, M-MDSC levels were negatively associated with peripheral CD8^+^ T cell expansion after ipilimumab treatment, and melanoma patients with lower basic M-MDSC levels (less than 14.9%) had a remarkably longer OS (Kitano et al., 2014).

The MDSCs-related factors causing ICIs resistance have also been investigated. In a longitudinal study on nivolumab, PMN-MDSCs were analyzed to decreased significantly in responders while those in non-responders did not show obvious change. In non-responsive patients, factors correlated with MDSCs recruitment and proliferation, including CXCL2, CCL23, C-X3-C motif chemokine ligand 1, and high mobility group box protein 1, were heavily aggregated (Kim et al., 2018). In patients with advanced melanoma, ipilimumab infusion could induce an elevated level of both M-MDSCs and relevant NO production in non-responders (Gebhardt et al., 2015). Furthermore, increased levels of lactate dehydrogenase (LDH) and IL-4Rα^+^ M-MDSCs after ipilimumab administration were associated with impaired OS in melanoma patients.

Given that the battle against cancer cells takes place mostly in tumor sites, the prevention of T cells infiltration or the dysfunction of infiltrated T cells pose more barriers. In order to figure out factors behind these two kinds of tumor immune inhibition, Jiang and colleagues developed a predictive gene signature, tumor immune dysfunction and exclusion. They demonstrated a correlation between MDSCs profiles and levels of CTLs and that MDSCs signature could predict reaction to anti-PD-1 and anti-CTLA-4 (Jiang et al., 2018). Additionally, myeloid-associated genes, such as cyclooxygenase-2, IL-8, IL-1β, in the tumor were associated with atezolizumab or durvalumab resistance in urothelial bladder cancer patients (Kim et al., 2015). In general, investigations on the role of tumor-derived MDSCs in resistance to ICIs is limited, hindering a clearer cognition on MDSCs and more effective combination immunotherapeutic strategies.

#### Effect on Adoptive T Cell Therapy

The ACT is another immunotherapy research hotspot. While several strategies modify T cells milieu, ACT directly infuses autologous or allogenic T cells. ACT separates and expands TILs and unmodified cytotoxic cells population from the resected tumor and then transfers them to patients to fight against tumor cells (Rosenberg and Restifo, 2015). TILs have been demonstrated positive data in malignant tumors, such as melanoma (Guedan et al., 2018; Chandran and Klebanoff, 2019). Lymphodepletion incorporation before TILs reinfusion increases response rate, with 20 of 92 patients in complete tumor regression, 19 of which do not relapse 3 years after treatment (Rosenberg et al., 2011). Compared with other modalities of ACT, these results highlight the superiority of pre-existing effector T cells with antitumor activities, which is not eligible in many cancer types (Perica et al., 2015).

T cells from peripheral blood equipped with CARs or TCRs have also revealed a noteworthy effectiveness in hematologic malignancy, with two CAR-T products (axicabtagene ciloleucel and tisagenlecleucel) approved by FDA. The first clinical application of optimized CD19-targeting CAR-T cells in CLL induced a significant response. Clinical trials of CD19-directed CAR T cells have also been conducted in B cell acute lymphoblastic leukemia and lymphoma, with a high complete remission rate (Brentjens et al., 2013; Park et al., 2018; Chong et al., 2021). TCRs-modified T cells targeting specific antigenic peptide-MHC complex expressed mainly in tumor cells are also effective in patients with solid tumors (Angelo et al., 2018). Bispecific T-cell engager (BiTE) is another ACT modality. BiTEs enhance the biological connection of T cells and tumor cells *via* two scFvs with separate close affinity to CD3 and tumor antigens (Thakur et al., 2018). Blinatumomab (MT103), the first FDA-approved BiTE, specific for CD3 and CD19, is used to treat several hematological malignancies. Actually, achievements of these ACT strategies were gained mostly in hematologic malignancies. Several ACT therapies targeting tumor-specific antigens or stroma-derived structures are under investigation, and only modest efficacy has been achieved in solid tumors. MDSCs-induced immunosuppressive microenvironment posed one of the critical obstinate hurdles.

Several studies have shown that MDSCs play dirty tricks on ACT therapy (Arina and Bronte, 2015; Fultang et al., 2019; Mengos et al., 2019). Besides autologous T cell suppression, MDSCs inhibit both the expansion and function of adoptively transferred T cells. A recent study reported that TILs infusion combined with lymphodepletion significantly increases CD11b^+^CD15^+^LOX-1^+^ PMN-MDSCs, which suppress TILs proliferation and IFN-γ production in melanoma and NSCLC patients (Innamarato et al., 2020). IL-6 was demonstrated to motivate hematopoietic progenitor cells after lymphodepleting. Subsequently, IL-6 and motivated hematopoietic progenitor cells promoted the generation and activation of MDSCs and inhibition of IL-6 enhanced response to ACT in mouse models. A preclinical rodent animal model study showed that CAR-T therapy increases MDSC levels in a GM-CSF-dependent manner, inhibiting the anti-tumor activity of adoptive T cells (Burga et al., 2015). IDO, an intracellular enzyme in MDSCs mediating tryptophan metabolism, hinders CAR-T therapy efficacy *via* tryptophan metabolites, and a tumor model showed that IDO inhibitor could restore the therapeutic effect (Fan et al., 2017).

Interestingly, while several studies revealed that MDSCs inhibit transferred T cell treatments, an animal experiment showed that MDSCs-co-cultured T cells augment ACT efficacy (Raber et al., 2016). Fewer T cells preconditioned with MDSCs differentiated into effector T cells before adoptive transferring, preserving anti-tumor capability. Inhibition of T cell differentiation is relied on cell-to-cell contact without hindering TCR function or early activation process. A well-designed CD33^–^ CAR-T cells, targeting both CD33^+^ blast and CD33^+^ MDSCs, promotes satisfied clinical results. The CD33xCD3 BiTEs studies reported similar results (Jitschin et al., 2018). Moreover, studies have reported that several MDSCs inhibition strategies enhance the anti-tumor effect of ACT therapy. However, the clinical trial outcomes are unknown (Fultang et al., 2019; Li et al., 2020; Sun et al., 2020).

#### Effect on Cancer Vaccines and Oncolytic Virus

Cancer vaccines and oncolytic virus enhance anti-tumor immune responses. Cancer vaccines contain several products, including immunocompetent cells, proteins, peptides, and nucleic acids, boosting T cell activation in tumors (Hu et al., 2018). The tumor-associated antigen (TAA) and neoantigen, mainly expressed in tumor cells, can be used in vaccine-based therapies (van der Bruggen et al., 2017). In contrast, oncolytic virus, modified viral particles, is an antigen-nonspecific agent used for cancer cell lysis to expose antigens, activating endogenous T cells to initiate cytotoxic responses to cancer cells (Kaufman et al., 2015). FDA has approved a DC vaccine product (sipuleucel T) and an oncolytic virus agent (talimogene laherparepvec) for prostate cancer and melanoma, respectively (Harrington et al., 2016; Kantoff et al., 2010). However, only a few neoantigen and TAAs can generate immune responses, possibly due to the negative selection during T cell development. To date, the clinical performance of cancer vaccines and the oncolytic virus is poor (Macedo et al., 2020).

Favorable outcomes of cancer vaccine and oncolytic virus treatment are dependent on decreased MDSC levels in both tumor models and cancer patients (Poschke et al., 2012; Laborde et al., 2014; Vandenberk et al., 2016). Several preclinical studies and clinical trials testing cancer vaccines and oncolytic virus efficacy have shown that high MDSC levels are related to poor response in cancer patients. Moreover, MDSCs levels increase after treatment (Clements et al., 2015; Keshavarz et al., 2020; Meng et al., 2020), indicating reinforced immunosuppression on anti-tumor immune response. A preclinical study on oncolytic vaccinia showed that increased PGE2 levels promote G-MDSC trafficking, inhibiting immunotherapeutic capability (Hou et al., 2016). Another study showed that the inflammatory component NLRP3, belonging to NOD-like receptor family, is essential in MDSCs-induced immunosuppression (van Deventer et al., 2010). In Nlrp3^–/–^ mice, few MDSCs reached the tumor site, and survival was fourfold in the DC vaccinated group than wild-type mice. However, removing MDSCs with anti-Gr-1 antibody was ineffective in Nlrp3^–/–^ mice, whereas it was effective in wild-type mice, suggesting that NLRP3 is essential in MDSC accumulation. Other studies also showed that MDSCs-derived molecules, including NO and TGF-β, inhibit vaccines and oncolytic virus treatment (Jia et al., 2010; Takaku et al., 2010). When MDSCs were significantly aggregated in the initial generating culture, DCs functions were significantly impaired (Poschke et al., 2012). A clinical trial investigating prophylactic vaccine (NCT02134925) showed high basic levels of circulating MDSCs, accounting for 22 poor responses of the 39 patients (Kimura et al., 2013). Generally, mounting evidence has shown that MDSCs negatively regulate the efficacy of cancer vaccines and oncolytic virus.

### Effect on Modulatory Cytokines

Cytokines administration is another strategy of immunotherapy and has been used as an adjuvant to modulate the immune system for robust anti-tumor immunity. T lymphocyte-promoting cytokines, including IL-2 and IFN-α, have gained significant advances. FDA first approved IL-2, an essential T cell growth factor, for cancer treatment. Clinical studies have revealed that it has generated a durable tumor regression in melanoma and renal cell carcinoma patients (Rosenberg, 2014). PEGylated IL-2 (bempegaldesleukin) combined with anti-PD-1 showed a 53% ORR in melanoma patients (Bentebibel et al., 2019). IFN-α is also an FDA-approved cytokine that induces the APC maturation to provide specific antigen presentation and costimulatory factors, triggering T cell activation and enhancing their cytotoxicity. However, IL-2 and other T cell activators have limited clinical utility, owing to their dose-limiting toxicities and facilitative effects on Tregs (Sim and Radvanyi, 2014; Floros and Tarhini, 2015). In recent years, immune-stimulatory cytokines have been mainly used in T cell expansion, promoting adoptive T cell therapies (Rosenberg, 2011).

Therapeutic cytokines are involved in the Th1 immune response, directly activating CTLs or enhancing activity of CTLs-promoting immune cells. However, several studies have shown that they have opposite effects on MDSCs during anti-tumor immune modulation. Alves et al. (2020) reported that there are more G-MDSCs (21.3%) in tyrosine kinase inhibitors (TKIs) and IFN-α combination therapy-treated patients than TKI treated patients (less than 10%), thus a poor immunosuppressive state. IL-2 also shows contradictory effects on PMN-MDSCs. Increased IL-2 levels extend MDSC lifespan in a dose-relevant manner, starting a backfire and impeding better therapeutic efficacy. Besides, augmented activated T cells and their increased GM-CSF production amplify the effect (Bauswein et al., 2018). Another study reported that IL-2 administration prevented the apoptosis of MDSCs and prolonged their survival, augmenting the destructive capability this cluster of cells (Pericle et al., 1994). To date, the effect of cytokines on immunotherapy is unclear. Further investigations are needed for the cytokine efficacy on malignant tumors and their regulation on the TME.

## MDSCs Targeting Improves T Cell-Based Immunotherapy Efficacy

**FIGURE 2:**
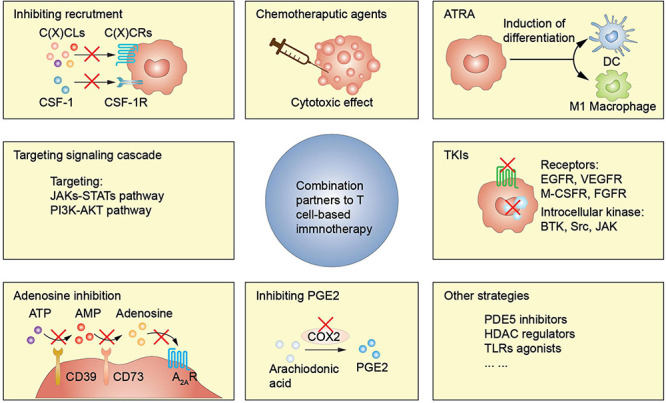
MDSCs-targeting strategies enhance T cell-based immunotherapy efficacy. ATRA, all-trans-retinoic acid; TKI, tyrosine kinase inhibitor; PDE5, phosphodiesterase-5; HDAC, histone deacetylase; TLRs, toll-like receptors.

It is necessary to combine T cell-based immunotherapy with MDSCs targeting agents since MDSCs are key players in immunosuppressive TME (Figure 2). Multiple studies have been conducted to investigate the effectiveness of relevant combined approaches (Table 2). In this section, we elaborate the main MDSCs-manipulating strategies employed to reinforce the antitumor activity of T cell-based immunotherapy, through inhibiting expansion and recruitment, promoting differentiation, inhibiting function, inhibiting metabolism, or deleting MDSCs directly.

**TABLE 2:** Combination strategies in clinical trials.

**MDSCs targets**	**MDSCs-targeting agents**	**Immunotherapy**	**Immunotheraputic agents**	**Indications**	**Phase**	**Last reported status**	**NCT number**
CSF-1R	ARRY-382	ICI	Pembrolizumab	Advanced solid tumors	I/II	Completed	NCT02880371
CXCR1/2	SX-682	ICI	Nivolumab	CRC	I/II	Recruiting	NCT04599140
CXCR1/2	Navarixin	ICI	Pembrolizumab	Solid tumors	II	Active, not recruiting	NCT03473925
CXCR2, chemotherapy	AZD5069, gemcitabine, nab-paclitaxel	ICI	MEDI4736	PDAC	I/II	Completed	NCT02583477
IL-8	BMS-986253	ICI	Nivolumab	Cancer	I/II	Active, not recruiting	NCT03400332
CCR2/CCR5, chemotherapy	BMS-813160, gemcitabine, nab-paclitaxel	ICI	Nivolumab	PDAC	I/II	Recruiting	NCT03496662
CCR2/CCR5, chemotherapy	BMS-813160, gemcitabine 5-fluorouracil	ICI	Nivolumab	CRC, pancreatic cancer	I/II	Recruiting	NCT03184870
CCR2/CCR5, IL-8	BMS-813160, BMS-986253	ICI	Nivolumab	NSCLC, HCC	II	Recruiting	NCT04123379
CCR2/CCR5, radiotherapy	CCR2/CCR5 dual antagonist, SBRT	ICI, vaccine	Nivolumab, GVAX	PDAC	I/II	Recruiting	NCT03767582
CCR5	Vicriviroc	ICI	Pembrolizumab	Colorectal neoplasms	II	Active, not recruiting	NCT03631407
Chemotherapy	Gemcitabine, fluorouracil, oxaliplatin	Cytokine	Aldesleukin, sargramostim	Pancreatic cancer	I/II	Active, not recruiting	NCT02620865
Chemotherapy	Cyclophosphamide	Cancer vaccine	IMA970A plus CV8102	HCC	I/II	Completed	NCT03203005
Chemotherapy	Hydroxychloroquine	Cytokine	IL-2	RCC	I/II	Completed	NCT01550367
Chemotherapy	Standard of care chemotherapy	ACT	Anti-CD3 x anti-EGFR bispecific antibody (EGFRBi) armed activated T cells (EGFR BATs)	Pancreatic adenocarcinoma	I/II	Recruiting	NCT03269526
Chemotherapy	Vinorelbine	ICI	Atezolizumab	NSCLC	II	Active, not recruiting	NCT03801304
Chemotherapy	Docetaxel	Cancer vaccine	mRNA transfected DCs	Prostatic neoplasms	II	Completed	NCT01446731
Chemotherapy	Doxorubicin, cyclophosphamide, paclitaxel	ICI	Pembrolizumab	BC	II	Recruiting	NCT02957968
Chemotherapy	Cyclophosphamide	Cytokine	Human recombinated IL-2	HCC	II/III	Recruiting	NCT04011033
Chemotherapy, radiotherapy	Fluorouracil radiation therapy	ICI	Avelumab	Genitourinary neoplasms	II	Active, not recruiting	NCT03617913
Chemotherapy, radiotherapy	Cyclophosphamide, chemoradiotherapy	Cancer vaccine	Tecemotide	Rectal cancer	II	Completed	NCT01507103
Chemotherapy, radiotherapy	Capecitabine, external beam irradiation	ICI	Avelumab	CRC	II	Recruiting	NCT03854799
Chemotherapy, radiotherapy	Cyclophosphamide, radiation	ICI	Pembrolizumab	Gynecological cancer	II	Recruiting	NCT03192059
Chemotherapy, radiotherapy	Cyclophosphamide, irradiation	ACT	Peripheral blood transplant	Hematologic malignancy	III	Recruiting	NCT03480360
ATRA	ATRA	ICI	Pembrolizumab	Melanoma	I/II	Active, not recruiting	NCT03200847
ATRA	ATRA	ICI	Ipilimumab	Melanoma	II	Active, not recruiting	NCT02403778
ATRA, chemotherapy	ATRA, cyclophosphamide	Cancer vaccine	Cancer vaccine	Lung cancer	II	Completed	NCT00601796
ATRA, chemotherapy	ATRA, paclitaxel	Cancer vaccine	Ad.p53-DC vaccines	SCLC	II	Completed	NCT00617409
TKI	Regorafenib	ICI	Nivolumab	HCC	I/II	Recruiting	NCT04170556
MDSCs targets	MDSCs-targeting agents	Immunotherapy	Immunotheraputic agents	Indications	Phase	Last reported status	NCT number
TKI	Dasatinib	Cancer vaccine	DC vaccine	Metastatic melanoma	II	Completed	NCT01876212
VEGF, HDAC	Bevacizumab, entinostat	ICI	Atezolizumab	Metastatic cancer, renal cancer	I/II	Recruiting	NCT03024437
VEGFR	Cabozantinib	ICI	Ipilimumab, nivolumab	Neuroendocrine carcinoma	II	Recruiting	NCT04079712
VEGFR	Cabozantinib	ICI	Ipilimumab, nivolumab	Thyroid cancer	II	Recruiting	NCT03914300
EGFR	Cetuximab	Cytokine	Edodekin alfa	Head and neck cancer	I/II	Active, not recruiting	NCT01468896
PI3K, VEGF, chemotherapy	IPI-549, bevacizumab, nab-paclitaxel	ICI	Atezolizumab	BC, RCC	II	Recruiting	NCT03961698
Akt	Ipatasertib	ICI	Atezolizumab	Solid tumor	I/II	Recruiting	NCT03673787
STAT3	Danvatirsen	ICI	Durvalumab	Cancer	II	Active, not recruiting	NCT02983578
STAT3, CXCR2	AZD9150, AZD5069	ICI	MEDI4736, tremelimumab	Solid tumor	II	Active, not recruiting	NCT02499328
TLR3	Poly ICLC	Cancer vaccine	IMA 950	CNS tumor	I/II	Completed	NCT01920191
TLR3	Poly ICLC	Cancer vaccine	Cancer vaccine	NSCLC	I/II	Recruiting	NCT01720836
TLR9	CMP-001	ICI	Nivolumab	Melanoma, lymph node cancer	II	Active, not recruiting	NCT03618641
TLR9	CMP-001	ICI	Avelumab	Advanced cancer	II	Recruiting	NCT02554812
HDAC	Entinostat	ICI	Pembrolizumab	NSCLC, melanoma, CRC	I/II	Unknown	NCT02437136
HDAC	Entinostat	ICI	Nivolumab	Cholangiocarcinoma, pancreatic cancer	II	Active, not recruiting	NCT03250273
CD73	MEDI9447	ICI	Durvalumab, tremelilumab	Ovarian cancer	II	Recruiting	NCT03267589
CD73	Oleclumab	ICI	Durvalumab	Sarcoma	II	Recruiting	NCT04668300
CD73, chemotherapy	MEDI9447, paclitaxel carboplatin	ICI	MEDI4736	TNBC	I/II	Recruiting	NCT03616886
IDO1	Epacadostat	ICI	Pembrolizumab	Melanoma	III	Completed	NCT02752074
MEK	Cobimetinib	ICI	Atezolizumab	GC, cholangiocarcinoma	II	Active, not recruiting	NCT03201458
Nrf2	Omaveloxolone	ICI	Ipilimumab, nivolumab	Melanoma	I/II	Completed	NCT02259231

*ICI, immune checkpoint inhibitor; ACT, adoptive T cell therapy; SBRT, stereotactic body radiation; CRC, colorectal cancer; PDAC, pancreatic ductal adenocarcinoma; NSCLC, non-small cell lung cancer; HCC, hepatocellular carcinoma; RCC, renal cell carcinoma; BC, breast cancer; SCLC, small cell lung cancer; CNS tumor, central nervous system tumor; TNBC, triple negative breast cancer; GC, gallbladder carcinoma.*

### Inhibiting Expansion and Recruitment

Blockers or antagonists of chemoattractants and their receptors could effectively diminish proportion of MDSCs both in circulation and TME, modifying the immunosuppressive microenvironment. The CXCLs-CXCR2 axis is essential in PMN-MDSC recruitment, and blocking the CXCLs-CXCR2 axis pathway inhibits PMN-MDSC aggregation (Highfill et al., 2014; Liao et al., 2019; Horn et al., 2020; Yang et al., 2021). CXCR2^+^ PMN-MDSCs also increase the expression of inhibitory immune checkpoints, such as PD-1, CTLA-4, and LAG3 on T lymphocytes, promoting T cells anergy (Zhu et al., 2017). Preclinical studies have reported that CXCR2 inhibitors or anti-CXCR2 antibodies alleviate MDSCs-induced immunosuppression, thus improving the effects of anti-PD-1 therapy in rhabdomyosarcoma, pancreatic ductal adenocarcinoma, and colorectal cancer (Steele et al., 2016; Horn et al., 2020). Clinical trials analyzing synergism in CXCR2 inhibitor SX-682 on pembrolizumab or nivolumab are underway (NCT03161431, NCT04477343, and NCT04599140). A human monoclonal antibody that inhibits one of its critical ligands, IL-8, has been used in phase I clinical trial (NCT02536469) (Bilusic et al., 2019) to interrupt CXCR2 pathway activation. Serum IL-8 level significantly reduced two days after anti-IL-8 administration. Anti-IL-8 and nivolumab efficacy is under clinical investigation (NCT03400332 and NCT04123379). CCR2 and CCR5 are essential in M-MDSC recruitment, and CCR5^+^ MDSCs enriched in tumor sites have a stronger immunosuppressive property. Flores-Toro et al. (2020) reported that CCR2 deficiency or administration of CCR2 antagonist CCX872 promotes efficacy of anti-PD-1 in mouse glioma model. CCX872 inhibits MDSC trafficking, increasing MDSC levels in the bone marrow and reducing their aggregation in the tumor site. The TILs data showed an increased population, elevated IFN-γ secretion, and decreased exhaustion marker expressions. Efficacy of CCR2/CCR5 dual inhibitor combined with nivolumab is under investigation (NCT03496662 and NCT03184870). CSF-1/CSF-1R pathway disruption also inhibits M-MDSCs infiltration, concomitantly influencing TAMs accumulation. Holmgaard et al. (2016) reported that CSF-1R expression on MDSCs is significantly increased after CTLA-4 blockade immunotherapy, with aggravated T cell inhibition. A combination of CTLA-4 blockade and anti-CSF-1R antibody promotes anti-tumor immunity and exacerbates tumor regression (Holmgaard et al., 2016). Additionally, selective CSF-1R targeting using specific inhibitors or antibody attenuates suppressive myeloid cells, and elective CSF-1R targeting combined with PD-1/PD-L1 blockade significantly control tumor growth in mouse neuroblastoma, breast cancer, and colorectal cancer models (Mao et al., 2016; Huang et al., 2020).

### Promoting Differentiation

All-trans-retinoic acid (ATRA), a member of retinoid family essential in differentiation induction and commonly used in acute promyelocytic leukemia treatment, also inhibits MDSC abundance. ATRA induces MDSC differentiation into macrophages and DCs *via* an ERK1/2 kinase signaling pathway (Bauer et al., 2018). A phase II clinical study showed that MDSC levels were significantly decreased in SCLC patients treated with combined P53-specific DC vaccine and ATRA compared with the DC vaccine alone (NCT00617409). The P53-specific response had a higher positive rate in the combination group, and granzyme B-positive CD8^+^ T cell enrichment was only observed in the combination group (Iclozan et al., 2013). Furthermore, ATRA synergistically enhanced ipilimumab efficacy in advanced melanoma patients (NCT02403778) (Tobin et al., 2018). In this study, MDSC levels increased in the ipilimumab monotherapy group and decreased in the combined treatment group. Moreover, HLA-DR^+^ myeloid cell levels significantly increased, promoting CD8^+^ T cell production, compared with ipilimumab treatment alone. Traditional chemotherapies inhibit MDSC trafficking and enable them to traffic back. In contrast, ATRA shows advantages by converting MDSCs into more differentiated cells, such as DCs and macrophages, essential in T cell-based immune response.

Toll-like receptors are transmembrane proteins that recognize protein or lipid ligands and activate transcription factors, facilitating the expression of pro-inflammatory factors, such as TNF-α and IL-2 (O’Neill et al., 2013). TLR7/TLR8 activation *via* TLR7/TLR8 agonist R848 induces M-MDSC differentiation into anti-tumor M1-type macrophages, whereas TLR1/TLR2 activation facilitates the M-MDSC transformation into suppressive M2-type macrophages (Wang et al., 2015; Shayan et al., 2018; Liu et al., 2020). B16 melanoma mouse model showed that R848-loaded b-cyclodextrin nanoparticles (CDNP-R848) improves anti-PD-1 treatment efficacy (Rodell et al., 2018). TLR3/TLR4 activation promotes receptor-interacting protein kinase 3 (RIPK3)-mediated programmed necrosis (Kearney and Martin, 2017). A RIPK3 reduction is associated with MDSC aggregation in colorectal cancer (Yan et al., 2018). Polyinosinic-polycytidylic acid (poly ICLC), TLR3 agonist, decreases MDSC levels, inhibiting their immunosuppressive function (Forghani and Waller, 2015). Additionally, poly ICLC significantly attenuates MDSC aggregation and function when combined with the CAR-T cells, enhancing cytotoxic activity of CAR-T cells *via* increased IL-2 and IFN-γ production (Forghani and Waller, 2015). TLR9 ligand CpG also blocks MDSC immunosuppression in T cells (Zoglmeier et al., 2011). CpG stimulation reduces Th2 cytokine and increases Th1 cytokine production in MDSCs and more M-MDSCs differentiate into tumoricidal M1 macrophages (Shirota et al., 2012). TLR7, TLR8, and TLR9 co-activation removes large tumors and builds an immune protective line by enhancing the NK cells and CTL infiltration, thus reducing MDSC levels (Zhao et al., 2014).

### Inhibiting Function

Signaling cascade-targeting agents compromising MDSCs differentiation, expansion, or function, including JAKs-STATs pathway, PI3K-AKT pathway, is an alternative to T cell-based immunotherapy. MDSCs functions, including immunosuppression and tumor promoting, are well orchestrated by STAT3 pathway. Strategies targeting STAT3 are under active investigation. A mouse liver metastatic tumor model showed that STAT3 inhibition enhanced the anti-tumor efficacy of CAR-T therapy by activating apoptotic signaling pathways and decreasing proliferative signaling pathways in liver-associated MDSCs (Guha et al., 2019). Clinical trials of STAT3 inhibition combined with ICIs are under investigation (NCT02983578 and NCT02499328). PI3K-AKT pathway is also essential in MDSC functions, migration, and metabolism (Martini et al., 2014). PI3Kγ, a PI3K subtype, promotes immunosuppression in malignancies and selectively targeting PI3Kγ with a specific inhibitor IPI-549 inhibits MDSCs-induced immunosuppression, restoring T cells-induced tumoricidal response (Kaneda et al., 2016). A mouse prostate cancer model engineered using signature mutations, BEZ235, a pan-class I PI3K/mTOR inhibitor, combined with immune checkpoint blockade showed a robust synergistic therapeutic efficacy (Lu et al., 2017a).

Several preclinical and clinical studies have shown that the phosphodiesterase-5 (PDE5) inhibitor, used to treat erectile dysfunction, inhibits MDSCs (Weed et al., 2019). A mouse tumor model showed that PDE5 inhibition with tadalafil attenuates MDSC suppressive capabilities by downregulating ARG1 and iNOS expressions, thus increasing T cell infiltration and activation (Yu et al., 2019). In the same study, administration of sildenafil was also approved to potentiate antitumor activity of adoptive therapy. In a clinical study, Tadalafil altered anti-tumor immunity in patients with recurrent HNSCC by downregulating MDSCs and Tregs and increasing cytotoxic CD8^+^ T cell levels in both peripheral blood and tumor site (Weed et al., 2019). Tadalafil combined with MUC1/polyICLC vaccine also inhibits PD-L1^+^ macrophage aggregation at the tumor edge, consistent with two clinical trials (NCT00843635 and NCT00894413) (Califano et al., 2015; Weed et al., 2015). However, the efficacy and survival of the trials investigating the role of a PDE5 inhibitor in immune modulation are unknown.

Entinostat, an epigenetic regulator, selectively targeting class I histone deacetylase (HDAC), inhibits MDSC function, reverses immune exclusion, and enhances anti-tumor activity. Entinostat combined with an epigenetic adjuvant, methyltransferase inhibitor 5-azacytidine, alters MDSC trafficking by downregulating CCR2 and CXCR2 expression, inducing MDSC differentiation into an interstitial macrophage-like phenotype (Lu et al., 2020). Murine breast cancer and pancreatic tumor models showed that entinostat combined with anti-PD-1 therapy or anti-CTLA-4 therapy significantly alleviates MDSCs-induced immunosuppression, increases activated granzyme-B-producing CD8^+^ T effector cell infiltration, significantly improving tumor-free survival (Christmas et al., 2018). A phase II clinical trial (NCT02437136) showed that entinostat combined with pembrolizumab has a favorable response (19%) and 36% clinical benefit rate in 53 progressed melanoma patients (Johnson et al., 2017). Besides, several HDAC inhibitors are being studied (Hashimoto et al., 2020; Kim et al., 2020).

### Inhibiting Metabolism

Adenosine also drives tumor progression *via* various mechanism, including MDSCs-mediated immunosuppression. Abrogating adenosine production by targeting nucleotide-metabolizing enzymes CD73 and CD39, as well as adenosine receptor A_2A_R seems to be a promising therapeutic strategy (Festag et al., 2020). A clinical study on ovarian cancer patients showed that metformin treatment downregulated the expression and catalytic activity of CD39 and CD73 in both M-MDSCs and PMN-MDSCs *via* adenosine monophosphate-activated protein kinase α (AMPKα) activation and HIF-1α suppression (Li L. et al., 2018). Besides, the circulating MDSC levels were decreased, and the cytotoxic activity of CD8^+^ T cells was restored, prolonging the OS of ovarian cancer patients with diabetes (Li L. et al., 2018). Additionally, anti-PD-1 immunotherapy upregulates CD73 level in melanoma patients (Reinhardt et al., 2017). A mouse breast cancer model showed that CD73 specific siRNA-loaded chitosan lactate nanoparticles improve tumor lysate pulsed DC vaccine efficacy. Furthermore, synergism is associated with MDSCs downregulation and T cells upregulation with reduced IL-10 levels and increased IFN-γ secretion (Jadidi-Niaragh et al., 2017). Several preclinical studies have shown that anti-CD73 therapy or A_2A_R targeting strategies significantly improves ICIs and ACT efficacy compared with monotherapy (Allard et al., 2013; Iannone et al., 2014; Mittal et al., 2014; Beavis et al., 2015; Hay et al., 2016; Reinhardt et al., 2017). Clinical trials evaluating the synergistic effects of CD73-A_2A_R targeting strategies and T cell-based therapy are underway.

Prostaglandin E2 is an inflammatory factor associated with carcinogenesis and MDSCs induction. COX-1 and COX-2 are key enzymes in PGE2 synthesis. COX-2/PGE2 signaling blockade inhibits MDSCs recruitment and represses MDSCs-induced immunosuppression, causing modified CTL cytotoxicity and enhanced tumoricidal immune response, thus improving therapeutic efficacy. Also, mounting evidence has shown that targeting PGE2 with non-steroidal anti-inflammatory drugs or specific COX-2 inhibitors, such as celecoxib, inhibiting MDSCs, improves immunotherapy outcomes (Veltman et al., 2010; Obermajer et al., 2011). A mouse melanoma model showed that licofelone, a dual COX/5-lipoxygenase (5-LOX) inhibitor, improves therapeutic vaccine efficacy by suppressing Gr-1^+^CD11b^+^ MDSC generation and minimizing IL-6 and IL-10 production (Neumann et al., 2016). These studies provide new insights for developing targeted COX-2-mediated PGE2 signaling combined with T cell-based immunotherapies.

### Deleting MDSCs

Several chemotherapeutic agents reduce MDSCs numbers, and combination strategies with immunotherapy can improve the survival of cancer patients (Draghiciu et al., 2015; Wang et al., 2017). Gemcitabine and 5-fluorouracil (5-FU) are among the most commonly used drugs to eliminate MDSCs in tumor models and cancer patients (Wang et al., 2017). A phase I/II study evaluated the efficacy of gemcitabine combined with pegintron (IFN-α) and p53 synthetic long peptide vaccine in ovarian cancer patients (NCT01639885) (Dijkgraaf et al., 2015). The study showed a significant reduce of MDSCs in gemcitabine group. Further, the combined therapy showed stronger vaccine-induced T-cell responses. In another study, Vincent et al. (2010) indicated that 5-FU selectively inhibits MDSCs, thus promoting T cell-based anti-tumor capability. However, various chemotherapeutic agents have different effects on MDSC modulation. A phase II clinical trial reported that the DC vaccine combined with docetaxel decreases MDSCs, which was an independent prognostic factor of disease-specific survival (NCT01446731) (Kongsted et al., 2017). Nonetheless, the combined therapy showed no significant clinical advantage over docetaxel monotherapy alone, possibly due to the limited included population. Moreover, a spontaneous melanoma model showed that 1 mg/kg paclitaxel administration reduces MDSC levels while 36 mg/kg dosage has no effect (Vincent et al., 2010; Sevko et al., 2013). Conversely, a 175 mg/m^2^ paclitaxel dose increased circulating MDSC levels (Diaz-Montero et al., 2009). Preconditioning EG7 tumor-bearing mice with a single low dose of doxorubicin or paclitaxel promoted ACT efficacy, with more activated and longer-sustained CD8^+^ T cells, probably due to MDSC inhibition *via* suppression of NF-κB and its associated immunosuppressive factors (Hsu et al., 2015). Ongoing clinical trials evaluating the synergistic effect of various chemotherapeutic agents combined with immunotherapy are shown in Table 2. Chemotherapeutic agents influence various cells and only MDSCs-related trials are included. There are many effects of chemotherapeutic agents on MDSC accumulation and various factors should be considered, including optimal drug combinations, administration dosage, intervals, and tumor types and stages.

Immunotherapy combined with TKIs provides an optional choice of standard treatment of several cancers (Hirsch et al., 2020). TKIs are commonly used agents in MDSCs manipulation. To target tyrosine kinases, sunitinib have been applied to patients with oligometastases of various cancer types. The progression-free survival and cause-specific survival were prolonged in sunitinib and radiotherapy combination arm due to the sunitinib-induced M-MDSC reduction and CD4^+^ and CD8^+^ T cell enrichment (Chen et al., 2015). However, a phase II clinical trial (NCT01118351) showed no improvement in non-muscle-invasive bladder cancer patients after sunitinib monotherapy regardless of the reversal of MDSCs-mediated immunosuppression (Zahoor et al., 2019). Preclinical or clinical studies have shown that several other TKIs, such as dasatinib and nilotinib, also inhibit MDSCs (Hughes et al., 2017). Notably, apart from MDSCs, TKIs influences other cells. For instance, sorafenib and dasatinib suppress T cells and NK cells in a dose-dependent manner, causing immunocompetence (Martin del Campo et al., 2015). VEGFR-TKI is a group of TKI that can be combined with T cell-based immunotherapy for its active role in angiogenesis and immune modulation. Antiangiogenic agents ameliorate TME by inhibiting immunosuppressive cell infiltration, including MDSCs and Tregs, and increasing recruitment of effector T cells and mature DCs.

High-dose radiation is also a candidate in MDSCs elimination. Radiotherapy is mainstay treatment for localized tumors and isolated metastasis, as well as patients with advanced cancer for palliative treatment. Radiotherapy accelerates immunogenic cell death and induces the release of tumor antigens and a cluster of inflammatory factors, including alarmins, cytokines, and chemokines (Weichselbaum et al., 2017). Certainly, these factors facilitate the infiltration of DCs, T cells, and MDSCs. cGAS-STING pathway is a critical signal in the infiltration of MDSCs and STING agonists alleviated MDSCs-mediated immunosuppression (Liang et al., 2017). The increased infiltration of MDSCs after radiotherapy was mostly witnessed in conventional fractionated radiation. However, ablative and/or hypofractionated radiation resulted the loss of MDSCs, leading to an enhanced antitumor immunity (Filatenkov et al., 2015; Lan et al., 2018). These reversed observations may be correlated with the earlier infiltration of cytotoxic CD8^+^ T cells (Deng et al., 2014; Filatenkov et al., 2015). When in combination with anti-PD-L1antibody, ablative hypofractionated radiation therapy was more potent for cancer treatment (Lan et al., 2018). Moreover, radiation could upregulate the expression of PD-L1. High-dose ionizing irradiation and PD-L1 blockade synergistically inhibited the infiltration of MDSCs, promoting antitumor immunity (Deng et al., 2014). Generally, radiotherapy is a promising strategy to enhance efficacy of T cell-based immunotherapy for its immune-stimulating capability. High-dose radiation has advantages in MDSCs elimination over conventional radiation.

## Conclusion

The last decade has witnessed an evolution in cancer treatment with the progress of immunotherapy. Scientists have expanded the understanding of cancer biology, identifying cytotoxic lymphocytes as a major force to combat against tumor cells. The personalized T cell-based immunotherapy has increasingly been used in cancer patients due to the increasing number of potential clinical trials and FDA-approved therapies. However, only a few patients benefit from these therapies. Mounting evidence suggests that T cell-based immunotherapy efficacy is associated with robust anti-tumor immune response, which is usually damaged in most cases. The fundamental goal of immunotherapy is infiltration of effector cytotoxic T cells. Nevertheless, immunosuppressive cells always work as a vital suppressive force of antitumor immune response. Among these populations, MDSCs play critical roles and pose a challenging to broader-spectrum benefits. Since ICIs are referred to as the “release the brakes” of the immune system, inhibition of MDSCs could act as effective brake pads and this may be an encouraging supplementary strategy for T cell-based immunotherapy. Rational combination therapies seem to be a promising resolution in cancer treatment.

Several therapeutic strategies combining MDSCs targeting strategies and T cell-based immunotherapy have been evaluated. However, most studies have failed, indicating that not all combination strategies synergistically enhance anti-tumor immunity. The immune system is complex, making it difficult to understand predictors. Moreover, MDSCs are a cluster of heterogeneous cells and it is difficult to target myeloid-derived cells due to their diversity, dynamic phenotypes, and functions. Therefore, it is necessary to further study the immunosuppressive network in the TME to reverse MDSCs-induced immunosuppression. To reveal a comprehensive landscape, multiomics and computer-assisted algorithms work more efficiently (Zhou, 2020). These discoveries and innovations will provide us a clear cognition on how to rationally design personalized integrative therapeutic strategies.

## Author Contributions

HS and KL drafted the manuscript. YN designed all figures and made substantial revision to the original manuscript. XZ and XL checked and modified the manuscript. All authors contributed to the article and approved the submitted version.

## Conflict of Interest

The authors declare that the research was conducted in the absence of any commercial or financial relationships that could be construed as a potential conflict of interest.
